# Supplementary material for: Phylogeographic History of Tomato Chlorosis Virus
Source: Viruses. 2025 Mar 22;17(4):457. doi: 10.3390/v17040457 (PMC12031228; doi:10.3390/v17040457)
Supplement: Supplementary file 1 [file viruses-17-00457-s001.zip › Table S2.pdf]

Table S2 Marginal likelihoods of different combinations of clock model and tree prior

Model of rate variation	Tree prior	Log marginal likelihood	
		Path Sampling	Step-stone sampling
Strict clock	Bayesian skyline	-4586.334	-4594.683
Strict clock	Constant size	-4609.886	-4611.775
Strict clock	Exponential growth	-4605.735	-4612.409
**Uncorrelated lognormal relaxed clock**	**Bayesian skyline**	**-4554.350**	**-4553.704**
Uncorrelated lognormal relaxed clock	Constant size	-4567.695	-4571.259
Uncorrelated lognormal relaxed clock	Exponential growth	-4564.604	-4565.534

The best-fitting tree prior and molecular clock model are indicated in bold font.
