# Supplementary figures and images for: Phylogeographic History of Tomato Chlorosis Virus
Source: Viruses. 2025 Mar 22;17(4):457. doi: 10.3390/v17040457 (PMC12031228; doi:10.3390/v17040457)

Fig. S1

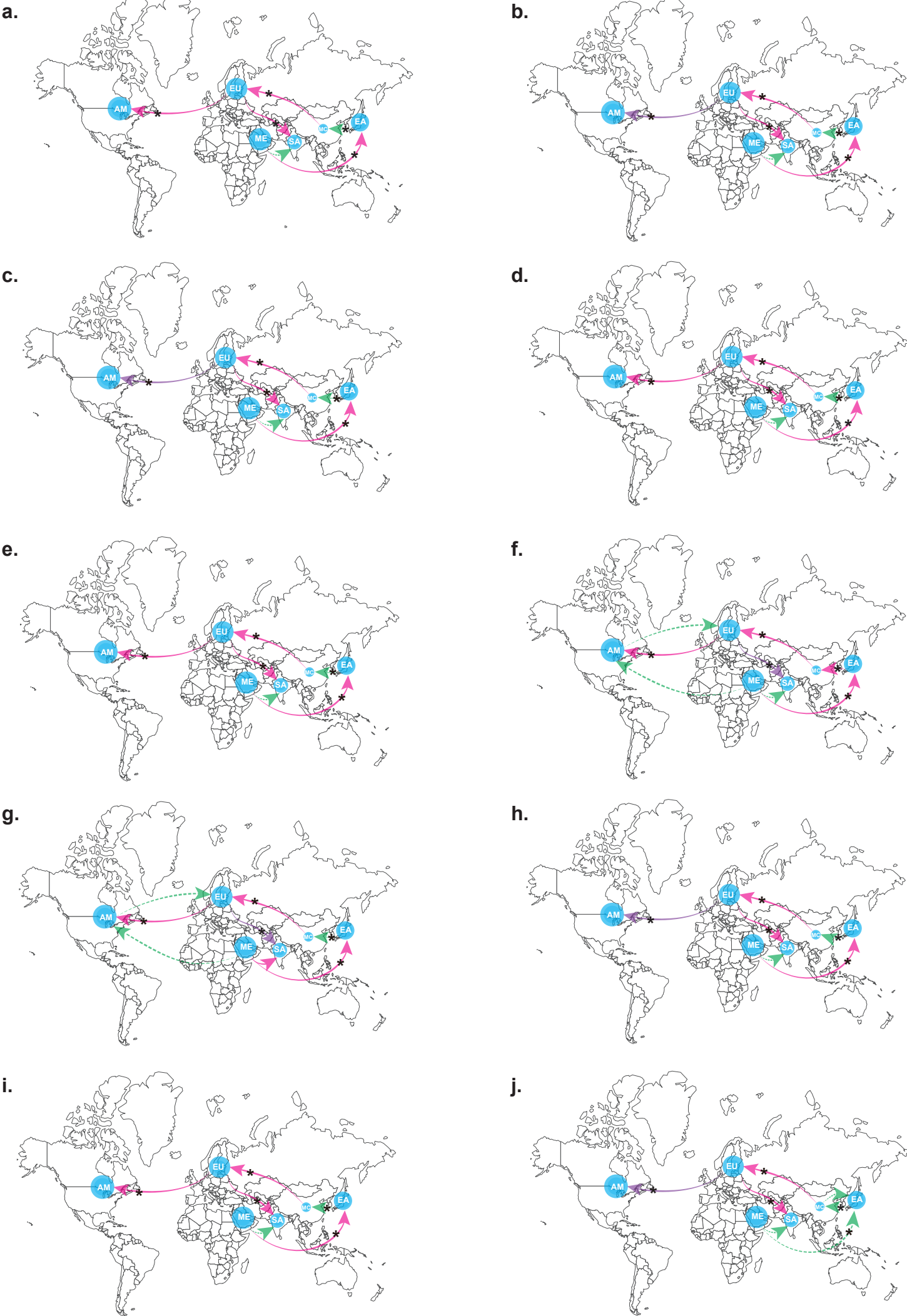

Supplement: Supplementary file 1 [file viruses-17-00457-s001.zip › Fig.S1.pdf]
